# Comparison of the use of ventricular access devices and ventriculosubgaleal shunts in posthaemorrhagic hydrocephalus: systematic review and meta-analysis

**DOI:** 10.1007/s00381-015-2951-8

**Published:** 2015-11-11

**Authors:** Daniel M. Fountain, Aswin Chari, Dominic Allen, Greg James

**Affiliations:** School of Clinical Medicine, University of Cambridge, Cambridge, UK; Victor Horsley Department of Neurosurgery, National Hospital for Neurology and Neurosurgery, London, UK; Division of Brain Sciences, Faculty of Medicine, Imperial College London, 5th Floor, Burlington Danes Building, Du Cane Road, London, W12 0NN UK; School of Medicine, Imperial College London, London, UK; Department of Neurosurgery, Great Ormond Street Hospital, London, UK; Developmental Neurosciences Programme, Institute of Child Health, University College London, London, UK

**Keywords:** Intraventricular haemorrhage, Posthaemorrhagic hydrocephalus, Ventricular access device, Ventriculosubgaleal shunt

## Abstract

**Introduction:**

Ventricular access devices (VAD) and ventriculosubgaleal shunts (VSGS) are currently both used as temporising devices to affect CSF drainage in neonatal posthaemorrhagic hydrocephalus (PHH), without clear evidence of superiority of either procedure. In this systematic review and meta-analysis, we compared the VSGS and VAD regarding complication rates, ventriculoperitoneal shunt conversion and infection rates, and mortality and long-term disability.

**Methods:**

The review was registered with the PROSPERO international prospective register of systematic reviews (registration number CRD42015019750) and was conducted in accordance with PRISMA guidelines.

**Results and conclusions:**

The literature search of five databases identified 338 publications, of which 5 met the inclusion criteria. All were retrospective cohort studies (evidence class 3b and 4). A significantly lower proportion of patients with a VSGS required CSF tapping compared to patients with a VAD (log OR −4.43, 95 % CI −6.14 to −2.72). No other significant differences between the VAD and VSGS were identified in their rates of infection (log OR 0.03, 95 % CI −0.77 to 0.84), obstruction (log OR 1.25, 95 % CI −0.21 to 2.71), ventriculoperitoneal shunt dependence (log OR −0.06, 95 % CI −0.93 to 0.82), subsequent shunt infection (log OR 0.23, 95 % CI −0.61 to 1.06), mortality (log OR 0.37, 95 % CI −0.95 to 1.70) or long-term disability (*p* = 0.9). In all studies, there was a lack of standardised criteria, variations between surgeons in heterogeneous cohorts of limited sample size and a lack of neurodevelopmental follow-up. This affirms the importance of an ongoing multicentre, prospective pilot study comparing these two temporising procedures to enable a more robust comparison.

**Electronic supplementary material:**

The online version of this article (doi:10.1007/s00381-015-2951-8) contains supplementary material, which is available to authorized users.

## Introduction

Preterm infants, particularly those classed as “extremely low birth weight” (<1000 kg), are at risk of bleeding from the germinal matrix of the developing brain, resulting in intraventricular haemorrhage (IVH). Depending on the grade of IVH, 25–80 % of affected infants develop radiological posthaemorrhagic ventricular dilatation (PHVD) and clinical evidence of posthaemorrhagic hydrocephalus (PHH) [1]. Preterm IVH is an important clinical problem in these children: PHH has been associated with significantly impaired long-term neurodevelopment [2]. PHH has been shown to result in a three-fold increase in cognitive and psychomotor delay, with nearly a third of patients suffering from epilepsy [3]. Furthermore, IVH is an independent risk factor for cerebral palsy [4, 5].

The definitive treatment for PHH is CSF diversion achieved by insertion of a ventriculoperitoneal shunt (VPS). Insertion of VPS is discouraged in infants weighing less than 2 kg due to immunological immaturity, technical factors and the risk of abdominal sepsis [6, 7]. They may also often have significant co-morbidities, including sepsis, respiratory impairment and abdominal complications such as necrotising enterocolitis [4]. In addition, a proportion of infants may not require permanent CSF drainage after clearance of the intraventricular blood [8, 9]. Therefore, in the interim, progressive symptomatic ventricular dilatation is often treated with temporary CSF diversion, with measures including lumbar punctures, ventricular tapping, external ventricular drains (EVDs) and the so-called temporising devices (TDs—see below). In previous systematic analyses, early repeated CSF tapping using lumbar punctures and ventricular tapping could not be recommended; lumbar punctures did not change outcomes in comparison to observation [10, 11]. The two most well-established TDs are a ventricular access device (VAD) and a ventriculosubgaleal shunt (VSGS). A VAD involves insertion of a subcutaneous reservoir connected to a ventricular catheter for percutaneous CSF tapping [12]; typically, these are aspirated percutaneously at regular intervals (e.g. every 12–48 h) in order to maintain head circumference [13]. A VSGS is a CSF shunt with the ventricular catheter draining directly into a subgaleal scalp pocket, created during the surgical procedure [4, 14]. There is evidence that VADs reduce morbidity and mortality compared with EVDs [11].

There are theoretical advantages in using a VSGS instead of a VAD; the VSGS permits resorption through a subgaleal scalp pocket, reducing the need for intermittent tapping required with a VAD [12, 14]. The VSGS also establishes a permanent decompression without causing electrolyte and nutritional losses [15]. However, potential complications of the use of a VSGS include scarring of the subgaleal pocket [14, 16], and/or CSF leakage [16–18], and some series report significant infection and failure rates [17, 19].

Currently, there is no strong evidence favouring the use of one particular TD over another, and the choice of which to use in a particular case is often down to the experience and preference of the treating neurosurgeon. Differences in outcomes between VADs and VSGSs as for the management of PHH remain poorly understood [11]. Therefore, this systematic review sought to compare VAD and VSGS in key outcomes such as complication rate (including infection and failure), permanent VPS requirement rate, long-term disability and mortality in neonates with PHH.

## Methods

This systematic review was conducted according to the PRISMA (Preferred Reporting Items for Systematic Reviews and Meta-Analyses) guidelines and has been registered with the PROSPERO international prospective register of systematic reviews (registration number CRD42015019750). A systematic search of keywords in Table 1 was performed independently by two authors (DMF and DA) of MEDLINE Complete via EBSCOhost, EMBASE 1974 to 2015 via Ovid, the Cochrane Central Register of Controlled Trials (CENTRAL) via the Cochrane Library and ClinicalTrials.gov databases on the 22nd April 2015. A record of our MEDLINE Complete search is provided in the Electronic Supplementary Material (Table S1).

**Table 1:** Search terms used in the literature review

Population	Problem	Intervention
Infant	Hemorrhage	Ommaya Reservoir*
Infant, Newborn	Hydrocephalus	VAD
Infant*	Hemorrhag*	Ventricular Access Device*
Neonat*	Haemorrhag*	Ventricular Reservoir*
	Intraventricular	Subcutaneous Reservoir*
	Intra Ventricular	VSGS
	Posthemorrhagic	Subgaleal Shunt*
	Posthaemorrhagic	Ventriculosubgaleal Shunt*
	Post Hemorrhagic	
	Post Haemorrhagic	

Medical Subject Headings (MeSH) are shaded in grey. Booleans “OR” and “AND” were utilised to combine row and column terms, respectively

Initially, titles and abstracts were screened for relevant papers. The full texts were then attained and reviewed. Both processes were undertaken by two authors independently (DMF and DA). Decisions were blinded and, where disagreements occurred, both authors discussed the disparities and resolved them throughout the selection process. Data extraction was also performed by two authors (DMF and DA) to ensure reliability. Where inconsistent reporting formats of data were published, authors were contacted directly to enable collection of comparable data using a standardised data collection template (Electronic Supplementary Material Table S2). The inclusion criteria were as follows:

1. Study design: Peer-reviewed published original research. Abstracts, commentaries, reviews and research without peer review were excluded.
2. Population: At least 10 patients with posthaemorrhagic hydrocephalus of prematurity (PHH).
3. Intervention: Use of ventricular access device (VAD) and ventriculosubgaleal shunt (VSGS) in a comparative study.
4. Outcome: Results including at least one of the following:
  - Rate of TD infection and obstruction
  - VPS conversion rate
  - Subsequent VPS infection
  - Mortality.

Evidence classification for accepted studies was performed based on the Oxford Centre for Evidence-based Medicine—Levels of Evidence [20]. All studies were appraised for their quality of reporting using the STROBE statement alongside separate evaluation of methods and validity of conclusions [21]. Where reported, data on each major outcome was converted to a log odds ratio (OR) with 95 % confidence interval (CI) and combined across studies in a meta-analysis. Reporting of meta-analysis was undertaken in accordance with the proposed checklist published by the Meta-analysis Of Observational Studies in Epidemiology (MOOSE) group [22]. Pooling of OR estimates was performed using an inverse variance weight random-effects DerSimonian-Laird meta-analysis, with Cochran’s *Q* test for heterogeneity [23]. If the *Q* test value was less than (*k*-1), where *k* = number of studies, fixed-effects method was also reviewed for consistency of results as a sensitivity analysis. All statistical analysis was performed using the *metafor* package in R, version 3.0.2 [24, 25].

A flow diagram for the results of the systematic search process is provided in the Electronic Supplementary Material (Figure S1). Forward and backward searching of accepted papers was also performed to identify additional studies not captured in the systematic search results. The search was repeated on 19th August 2015. No additional studies were identified.

## Results

**Fig. 1:**
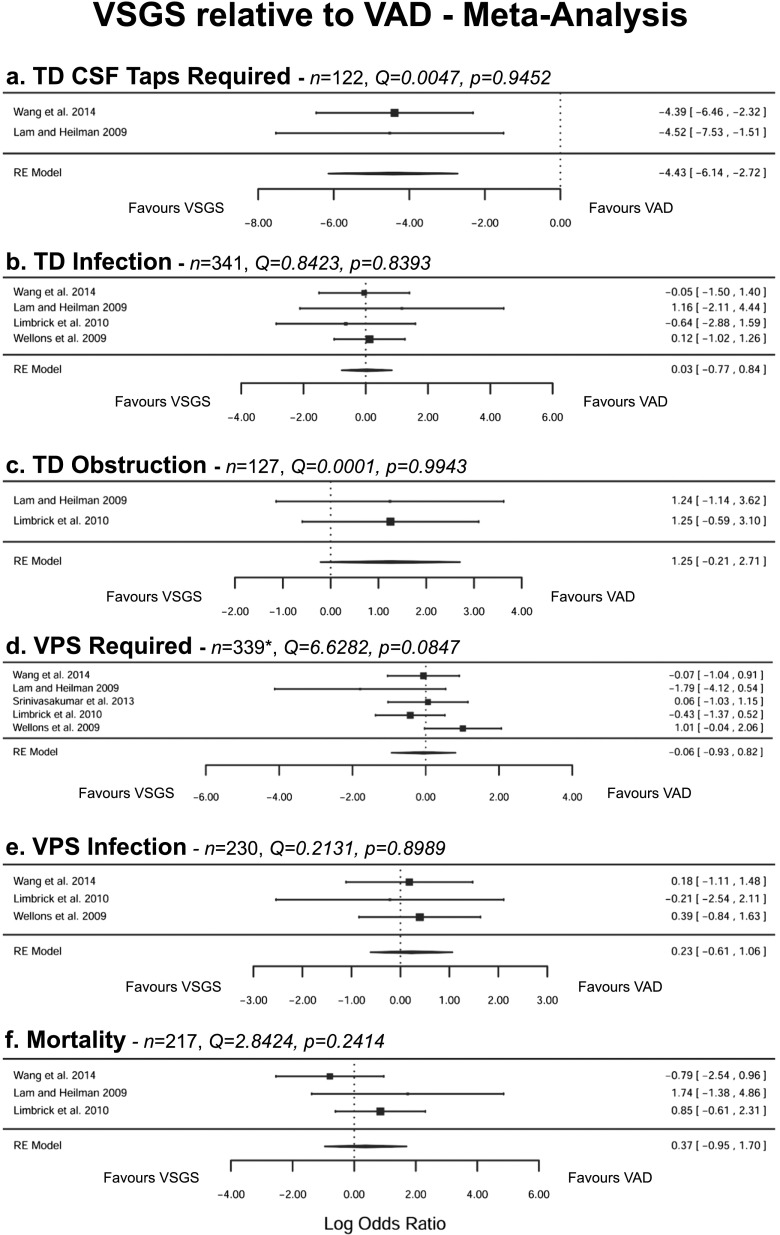
Meta-analysis of VSGS relative to VAD. Statistics presented are Cochran’s *Q* with *p* value test for heterogeneity and log odds ratio (OR) with 95 % confidence intervals (CI). An *asterisk* represents the results for VPS conversion rate from Srinivasakumar et al. [26] excluded due to potential overlap with Limbrick et al. [27]

Five original research studies were identified, all retrospective cohort studies (Table 2). Three studies [8, 26, 27] were classified as class 3b due to tested homogeneity of birth weight and gestational age between cohorts. Wang et al. tested the cohorts and found them to be significantly heterogeneous, whereas Wellons et al. conducted no testing for homogeneity of cohorts [28, 29]. Both studies were thus assigned as evidence class 4. Meta-analysis of the published data where pooling was possible is presented in Fig.1. Across all outcome measures, Cochran’s Q test for heterogeneity was non-significant (*p* > 0.05). Fixed effect sensitivity analysis provided consistent results where performed.

**Table 2:** Literature search results

Citation	Type of study	Evidence class	Sample/*n*	Defined outcome(s)	Results	STROBE score (/34)*	Comments
Wang et al. [26]	Retrospective cohort	4	VAD = 44	1. Number of TD CSF taps	VAD insertion was predictive of more CSF taps prior to VPS placement compared with VSGS placement (10 ± 8.7 taps vs 1.6 ± 1.7 taps, *p* < 0.001). No significant differences in the rates of TD infection requiring removal (VAD 6.8 %, VSGS 6.5 %, *p* = 0.96), VPS insertion (VAD 77.3 %, VSGS 76.1 %, *p* = 0.89) or early VPS infection (VAD 11.4 %, VSGS 13.0 %, *p* = 0.78)	28	VAD data collected 1998–2007, VSGS data collected 2008–2011 following switch due to theorised benefit. Possibility of selection bias. Incomplete VAD follow-up data and imaging. No long-term neurodevelopmental outcomes. Significant differences in gestational age and mean weight between the two groups
			VSGS = 46				
			Total = 90	2. TD infection			
				3. VPS conversion			
				4. VPS infection			
Lam and Heilman [6]	Retrospective cohort	3b	VAD = 16	1. Need for CSF taps	16/16 patients with VAD required daily taps while 4/16 patients with VSGS required daily taps (*p* = 0.000016). 1 obstruction was found in the VAD group compared to 3 obstructions in the VSGS group (*p* = 0.17). 1 CSF infection was found in the VSGS group. 15/16 (93.8 %) of patients with VAD required a permanent VPS compared to 10/14 (71.4 %) patients with VSGS. Two patients with VSGS died from complications unrelated to the TD surgery	22	No significant difference between the mean gestational age and mean birth weight of the VAD and VSGSS groups. No long-term neurodevelopmental outcomes
			VSGS = 16	2. TD infection			
			Total = 32	3. TD obstruction			
				4. VPS conversion			
Srinivasakumar et al. [24]	Retrospective cohort	3b	VAD = 29	1. Early vs. late intervention	17/29 (58.5 %) with VAD versus 15/25 (60.0 %) with VSGS underwent VPS placement (*p* = 0.7). Interventions were comparable with respect to mortality rate (*p* = 0.8), infection rate (*p* = 0.3), revision rate (*p* = 0.9), follow-up (*p* = 0.9), and neurodevelopmental outcome (*p* = 0.9) at 18 to 24 months	31	Limited follow-up information available. Lack of neuroimaging to determine the impact of white matter injury. Not possible to ascertain overlap with similar study from same centre
			VSGS = 25				
			Total = 54	2. VPS conversion			
				3. Neurodevelopmental outcome			
Limbrick et al. [25]	Retrospective cohort	3b	VAD = 65	1. TD infection	49 (75.4 %) patients with VAD and 20 (66.7 %) patients with VSGS required VPS s (*p* = 0.38). No statistical difference between VAD or VSGS with regard to device infection (VAD 6.2 %, VSGS 3.3 %, *p* = 0.57), need for revision (VAD 3.1 %, VSGS 10 %, *p* = 0.16), subsequent VPS infection (VAD 4.6 %, VSGS 3.3 %, *p* = 0.77), VPS revision rate (*p* = 0.58) or mortality rate (VAD 6.2 %, VSGS 13.3 %, *p* = 0.24)	29	Single institution study of only six surgeons. Lack of criteria for timing of TD, creating inherent variability and confounders. No long-term neurodevelopmental outcomes
			VSGS = 30	2. TD obstruction			
			Total = 95	3. VPS conversion			
				4. VPS infection			
				5. VPS revision			
				6. Mortality rate			
Wellons et al. [27]	Retrospective cohort	4	VAD = 88	1. VPS conversion	61/88 (69.3 %) patients who received VAD and 31/36 (86.1 %) patients who received VSGS also received permanent CSF diversion with a VPS (*p* = 0.05). 11 patients with VAD (13 %) had CSF infections, compared with 5 patients (14 %) in the VSGS group (*p* = 0.83). 6-month incidence of permanent shunt infection in patients with VAD was 7/61 (12 %), compared with 5/31 (16 %) of patients with VSGS (*p* = 0.65)	30	Four centres involved each with different criteria for initial treatment. Criteria for use of TD not standardised and only two centres performed it. Variations between surgeons in each centre were also present. No long-term neurodevelopmental outcomes
			VSGS = 36	2. TD infection			
			Total = 124	3. VPS conversion			
				4. VPS infection rate at 6 months			

*TD* temporising device, *VPS* ventriculoperitoneal shunt

*STROBE score out of 33 for studies with no missing data

### Temporising device CSF taps, infection and obstruction

Two studies evaluated CSF tapping. Wang et al. identified a significantly higher number of CSF taps in patients with VADs than VSGSs (VAD 10 ± 8.7 taps vs. VSGS 1.6 ± 1.7 taps, *p* < 0.001) [28]. This was the case despite a significantly longer time from TD to VPS (VAD 48.8 ± 26.4 days vs. VSGS 80.8 ± 67.5 days, *p* = 0.012). Furthermore, Lam and Heilman reported that 16/16 patients with VADs required daily CSF tapping, compared to only 4/16 patients with VSGSs (*p* = 0.000016) [8]. Meta-analysis showed a significantly lower proportion of patients with a VSGS requiring CSF tapping compared to patients with a VAD (Fig. 1a, log OR −4.43, 95 % CI −6.14 to −2.72). However, this did not translate to significantly higher rates of infection or obstruction with VADs compared to VSGSs in either study. Infection rates reported in four studies ranged from 0 to 12.5 % for VADs and 6.3 to 13.9 % for VSGSs [8, 27–29]. Meta-analysis showed no significant difference between VAD and VSGS rates of infection and obstruction (Fig. 1b, c; infection log OR 0.03, 95 % CI −0.77 to 0.84; obstruction log OR 1.25, 95 % CI −0.21 to 2.71). One study investigated the need for revision, finding no significant difference between revision rates in patients with a VAD and VSGS (VAD 3.1 % vs. VSGS 10 %, *p* = 0.16) [27].

### Ventriculoperitoneal shunt dependence

The proportion of patients with a VAD and VSGS converted to VPS varied greatly between studies (58.5–93.8 % for VADs and 60–86 % for VSGSs). Whilst the Wellons et al. study found borderline significance in the number of patients with a VSGS requiring a VPS (69.3 % for VADs vs. 86.1 % for VSGSs, *p* = 0.05) [29], meta-analysis of the studies found no significant difference (Fig. 1d, log OR −0.06, 95 % CI −0.93 to 0.82). Subsequent VPS infection rates were comparable between patients with VADs (4.6–12 %) and VSGSs (3.3–16 %), with no significant difference following meta-analysis (Fig. 1e, log OR 0.23, 95 % −0.61 to 1.06).

### Mortality

Despite representing a core outcome in the management of posthaemorrhagic hydrocephalus and the preterm infant, only three studies reported mortality. In the studies, all mortalities were unrelated to the posthaemorrhagic hydrocephalus or complications relating to its management [8, 27, 28]. No significant difference in mortality was found between patients with VADs and VSGSs in the reporting studies and in meta-analysis of the data (Fig. 1f, log OR 0.37, 95 % CI −0.95 to 1.70).

### Long-term neurodevelopment

Only one study reported neurodevelopmental outcome. Srinivasakumar et al. utilised the Bayley Scales of Infant and Toddler development, with results at 18 and 24 months for 36 % of surviving infants. Comparative testing between patients with a VAD and VSGS was not significant (*p* = 0.9) [26].

### Criteria

Various criteria were reviewed relating to the decision to place a TD, the TD type, at which point(s) CSF tapping was indicated, when VPS conversion was indicated and patient inclusion in the studies (Table 3). TD placement criteria varied between studies, but common indications included clinical instability of the infant, identified PHVD or external signs of raised intracranial pressure. Two studies gave no detail relating to indications for TD placement. The decision on the type of TD was either due to surgeon training or preference, or the preference of the centre. Two studies evaluated centres that had transitioned all treatment from VAD to VSGS on the grounds of theorised benefits of the VSGS [8, 28].

**Table 3:** Criteria described in included studies

Citation	TD Placement	TD Type	CSF tapping	VPS Conversion	Study Inclusion/Exclusion Criteria
Wang et al. [26]	None given	Switch from VAD to VSGS in 2007 due to agreement amongst surgeons of theorised benefits	1. Vital sign instability	1. PPHVD on ultrasound	1. Treated with VAD 1998–2007, VSGS 2007–2011
			2. Rapid increase in head circumference	2. Rapid increase in head circumference	
					2. IVH and PHH diagnosed with CUS
			3. Increase in ventricular size by CUS	3. Vital sign instability	
				4. Weight > 2000 g	3. EGA < 37 weeks
Lam and Heilman [6]	None given	Switch from VAD to VSGS in 2002 to minimise need for daily taps and provide more decompression of ventricles	VAD: Daily CSF tapping from 1 day postoperatively, continued if hydrocephalus identified on CUS	Weight > 2500 g	1. Treated with VAD 1994–2002, VSGS 2002–2008
					2. Prematurity
			VSGS: When CSF absorption from subgaleal pocket no longer adequate		
Srinivasakumar et al. [24]	1. Presence of PPHVD on biweekly follow-up CUS	Surgeon preference	None given	1. Need for continued CSF tapping	1. EGA ≤ 34 weeks
					2. Grade III or IV IVH
	2. Clinical stability of the infant			2. EGA > 40 weeks	3. Infants with congenital central nervous system malformations and stroke excluded
				3. Weight > 2500 g	
Limbrick et al. [25]	1. PPHVD	1. Surgeon training	1. Timing and volume of CSF extraction in VAD and VSGS both neurosurgeon determined	1. Need for continued CSF tapping	1. Treated 1999–2008
	2. Increasing daily head circumference	2. Surgeon preference			2. EGA < 40 weeks
				2. EGA 40–44 weeks	3. Weight < 1500 g
	3. Tense anterior fontanelle		2. VSGS tapping performed following scarring of subgaleal pocket	3. Weight 1800–2000 g	4. Grade III or IV IVH
	4. Splaying of cranial sutures			4. Consensus neonatologist and neurosurgeon	5. Use of TD
	5. Change in neurological status or vital signs				
Wellons et al. [27]	Disparate between centres and surgeons but included:	Surgeon and centre preference	None given	Disparate between centres and surgeons.	1. Patients who died or received care at an outside facility excluded
	1. Head size and rate of growth			Permanent shunt placement without TD based on:	
	2. PPHVD				2. Birth 2001–2006
	3. Change in neurological status or vital signs			1. Adequate weight	3. Grade III or IV IVH
				2. Radiographic criteria for blood products within the ventricle on imaging	4. Weight < 1500 g
	4. Size and turgor of anterior fontanelle				5. Use of TD for PHH
	5. Degree of separation of skull sutures				

*TD* temporising device, *VPS* ventriculoperitoneal shunt, *PHH* posthaemorrhagic hydrocephalus, *CUS* cranial ultrasonography, *PHVD* posthaemorrhagic ventricular dilatation, *EGA* estimated gestational age

Indications for CSF tapping included instability of vital signs, rapid increase in head circumference, signs of hydrocephalus on cranial ultrasonography and failure of adequate absorption of the subgaleal pocket in patients with VSGSs. Whilst one study reported routine tapping until signs of hydrocephalus resolved [8], two studies indicated CSF tapping as required [27, 28]. The two remaining studies provided no clear details of CSF tapping protocols [26, 29]. For treatment of CSF infection, one study reported removing the device based on surgeon preference [28]. Specific VPS conversion criteria varied greatly between studies but included elements related to the weight and estimated gestational age (EGA) of the infant, vital sign deterioration, persisting PHVD and need for CSF tapping. Furthermore, criteria for patient inclusion in the study varied. Three studies specified patients with a diagnosed grade III or IV IVH [26, 27, 29]. Two studies specified a weight of less than 1500 g [27, 29]. Exclusion criteria included patients with congenital central nervous system malformations and stroke, and those who received care outside of the facility studied [26, 29]. Where specified, EGA for the patients included ranged from 34 to 40 weeks [26–28].

## Discussion

Identifying the optimal temporising device for PHH is important in limiting potentially devastating consequences from this complication of IVH. The decision to use either a VAD or VSGS remains poorly understood [11], and this is the first review to compare these temporising devices in a systematic way across multiple outcomes and meta-analyse the results where possible. Whilst criteria for diagnosing IVH were somewhat comparable in the characteristics reviewed, specific measures or thresholds for subsequent PPHVD were not reported in any included study. This finding corroborates a survey of neonatologists that demonstrated substantial heterogeneity of diagnosis and management of this condition across Europe [30].

For those studies that reported TD infection rates, two of the five studies included criteria for infection (CSF culture positivity) [27, 28]. Although CSF tapping has been associated with an increased risk of infection [10], the results here demonstrate that, despite a significantly higher rate of tapping in patients with a VAD, there is no evidence to suggest a higher rate of infection of the VAD relative to the VSGS. Despite the theoretical risks, it is hypothesised that the use of rigorous protocols for CSF tapping minimises the infection rate for VADs [31]. Methods of CSF tapping used to minimise infection were not described, but the range of infection rates reported suggests potential differences between centres in absolute infection rates.

The significantly reduced rate of tapping may be an attractive feature of VSGS for neurosurgical units in certain localities. Whilst in North America these infants are likely to stay at the neurosurgical centre for the majority of their neonatal care, where tapping of the VAD can be supervised and performed by neurosurgical professionals, many European centres will discharge these children soon after surgery to their local neonatal unit for ongoing care. The lack of “control” over frequency and technique of tapping in peripheral hospitals may push these units towards the use of VSGS. Standard protocols for indications for CSF tapping were not consistently used across studies, including the clinical indications, timing of tapping and volume of CSF extracted. Furthermore, standard protocols for the treatment of CSF infection or TD obstruction were not described in the studies included in this review.

VPS conversion and subsequent infection was not significantly different between patients managed with a VAD or VSGS, but this finding is confounded by the heterogeneity in timing and criteria used to decide when to convert to a permanent VPS; the time interval from TD to VPS was analysed in only one study [28]. Similar to the TD infection rate, the VPS infection rate varied greatly between studies. Protocols for management of VPS infections were not described in the studies included in this review.

With regard to mortality and neurodevelopmental status, there is potential selection bias as Wellons et al. excluded patients who died and Limbrick et al. reported substantial co-morbidities that contributed to the mortality rate [27, 29]. Furthermore, all of the aforementioned outcome measures are potential confounders, in particular TD and VPS infection. The length of follow-up in studies also varied greatly, with no standard criteria for the determination of a mortality rate reported. A multivariable model approach would better elucidate the factors contributing to long-term neurodevelopmental outcome.

The results affirm the importance of an ongoing multicentre, prospective pilot study comparing these two temporising procedures to enable a more robust comparison, with standardisation of protocols across diagnosis, TD insertion, VPS conversion and measurement of long-term outcome [32].

### Limitations

The absences of a rationale for the sample size, a participant flow diagram or the use of sensitivity analysis were key omissions that reduced the STROBE scores for the included studies. Major specific limitations identified in the studies include heterogeneity of patient cohorts [28], lack of neurodevelopmental follow-up [8, 27–29], absence of standardised criteria for the management of this condition and variations between surgeons creating inherent variability and confounders [27, 29]. Limited sample sizes reduced statistical power in all studies. The meta-analysis presented gives estimates across core outcomes to provide results from a larger sample. However, whilst heterogeneity was assessed statistically in the meta-analysis, it was nonetheless performed on a series of retrospective studies with substantial heterogeneity in the criteria for management and reporting of outcomes in patients with posthaemorrhagic hydrocephalus.

## Conclusion

This study reports, in an objective and systematic fashion, the current state of the literature regarding which TD (VAD or VSGS) is superior for the management of PHH in preterm infants. The systematic search revealed an absence of randomised controlled trials investigating this clinical equipoise. Five studies of poor quality (three class 3b studies and two class 4 studies) were identified and reviewed, with an observational study meta-analysis performed. There is evidence to suggest fewer CSF tappings are necessary with a ventriculosubgaleal shunt. Meta-analysis showed no significant differences between VAD and VSGS in rates of temporising device infection, obstruction, the requirements for a ventriculoperitoneal shunt, infection of the subsequent shunt and overall mortality. Heterogeneity of included studies reaffirms the importance of standardised criteria for initial management and reporting outcomes, along with a more systematic approach to sustained follow-up to enable a better understanding of long-term neurodevelopment of these patients. There is not current sufficient data to suggest superiority of one TD over the other, although the reduced rate of CSF tapping in VSGS may make this option attractive in certain healthcare systems.

## Electronic Supplementary Material

ESM 1 (DOCX 14.3 kb)

ESM 2 (DOCX 11 kb)

ESM 3 (DOCX 406 kb)
