# Supplementary material for: Comparison of the use of ventricular access devices and ventriculosubgaleal shunts in posthaemorrhagic hydrocephalus: systematic review and meta-analysis
Source: Childs Nerv Syst. 2015 Nov 11;32:259–67. doi: 10.1007/s00381-015-2951-8 (PMC4749661; doi:10.1007/s00381-015-2951-8)
Supplement: Supplementary file 1 — (DOCX 14.3 kb) [file 381_2015_2951_MOESM1_ESM.docx]

**Electronic Supplementary Material**

*Table S1 – MEDLINE Complete Search Strategy*

| **Search ID#** | **Search Terms** | **Search Options** | **Last Run Via** | **Results** |
| --- | --- | --- | --- | --- |
| S10 | S4 AND S8 AND S9 | Search modes - Boolean/Phrase | Interface - EBSCOhost Research Databases | 140 |
|  |  |  | Search Screen - Advanced Search |  |
|  |  |  | Database - MEDLINE Complete |  |
| S9 | Ommaya Reservoir* OR VAD OR Ventricular Access Device* OR Ventricular Reservoir* OR Subcutaneous Reservoir* OR VSGS OR Subgaleal Shunt* OR Ventriculosubgaleal Shunt* | Search modes - Boolean/Phrase | Interface - EBSCOhost Research Databases | 7,555 |
|  |  |  | Search Screen - Advanced Search |  |
|  |  |  | Database - MEDLINE Complete |  |
| S8 | S5 OR S6 OR S7 | Search modes - Boolean/Phrase | Interface - EBSCOhost Research Databases | 351,748 |
|  |  |  | Search Screen - Advanced Search |  |
|  |  |  | Database - MEDLINE Complete |  |
| S7 | Hemorrhage OR Hydrocephalus OR Hemorrhag* OR Haemorrhag* OR Hemorrhage OR Intraventricular OR  Intra Ventricular OR Posthemorrhagic OR Posthaemorrhagic OR Post Hemorrhagic OR Post Haemorrhagic | Search modes - Boolean/Phrase | Interface - EBSCOhost Research Databases | 351,748 |
|  |  |  | Search Screen - Advanced Search |  |
|  |  |  | Database - MEDLINE Complete |  |
| S6 | (MH "Hydrocephalus") | Search modes - Boolean/Phrase | Interface - EBSCOhost Research Databases | 18,514 |
|  |  |  | Search Screen - Advanced Search |  |
|  |  |  | Database - MEDLINE Complete |  |
| S5 | (MH "Hemorrhage") | Search modes - Boolean/Phrase | Interface - EBSCOhost Research Databases | 56,653 |
|  |  |  | Search Screen - Advanced Search |  |
|  |  |  | Database - MEDLINE Complete |  |
| S4 | S1 OR S2 OR S3 | Search modes - Boolean/Phrase | Interface - EBSCOhost Research Databases | 1,169,898 |
|  |  |  | Search Screen - Advanced Search |  |
|  |  |  | Database - MEDLINE Complete |  |
| S3 | Infant* or Neonat* | Search modes - Boolean/Phrase | Interface - EBSCOhost Research Databases | 1,169,898 |
|  |  |  | Search Screen - Advanced Search |  |
|  |  |  | Database - MEDLINE Complete |  |
| S2 | (MH "Infant, Newborn") | Search modes - Boolean/Phrase | Interface - EBSCOhost Research Databases | 504,413 |
|  |  |  | Search Screen - Advanced Search |  |
|  |  |  | Database - MEDLINE Complete |  |
| S1 | (MH "Infant") | Search modes - Boolean/Phrase | Interface - EBSCOhost Research Databases | 652,932 |
|  |  |  | Search Screen - Advanced Search |  |
|  |  |  | Database - MEDLINE Complete |  |
