# Supplementary material for: Comparison of the use of ventricular access devices and ventriculosubgaleal shunts in posthaemorrhagic hydrocephalus: systematic review and meta-analysis
Source: Childs Nerv Syst. 2015 Nov 11;32:259–67. doi: 10.1007/s00381-015-2951-8 (PMC4749661; doi:10.1007/s00381-015-2951-8)
Supplement: Supplementary file 2 — (DOCX 11 kb) [file 381_2015_2951_MOESM2_ESM.docx]

*Table S2 – Data collection template sent to all authors*

| **Measure** | **VAD** | **VSGS** |
| --- | --- | --- |
| *Total (n)* |  |  |
| *Male (n)* |  |  |
| *Mean gestational age in weeks (SD)* |  |  |
| *Mean birth weight in kg (SD)* |  |  |
| *IVH Grade III (n)* |  |  |
| *IVH Grade IV (n)* |  |  |
| *Infection (n)* |  |  |
| *CSF leak (n)* |  |  |
| *Blockage (n)* |  |  |
| *CSF taps required at any time (n)* |  |  |
| *Ventriculoperitoneal Shunt required (n)* |  |  |
| *Deceased (n)* |  |  |
| *Ventriculoperitoneal Shunt infections (n)* |  |  |
