# Supplementary material for: Comparison of the use of ventricular access devices and ventriculosubgaleal shunts in posthaemorrhagic hydrocephalus: systematic review and meta-analysis
Source: Childs Nerv Syst. 2015 Nov 11;32:259–67. doi: 10.1007/s00381-015-2951-8 (PMC4749661; doi:10.1007/s00381-015-2951-8)

*Figure S1 – Flow Diagram of Literature Search Process. VAD, ventricular access device; VSGS, ventriculosubgaleal shunt; PHH, posthaemorrhagic hydrocephalus*


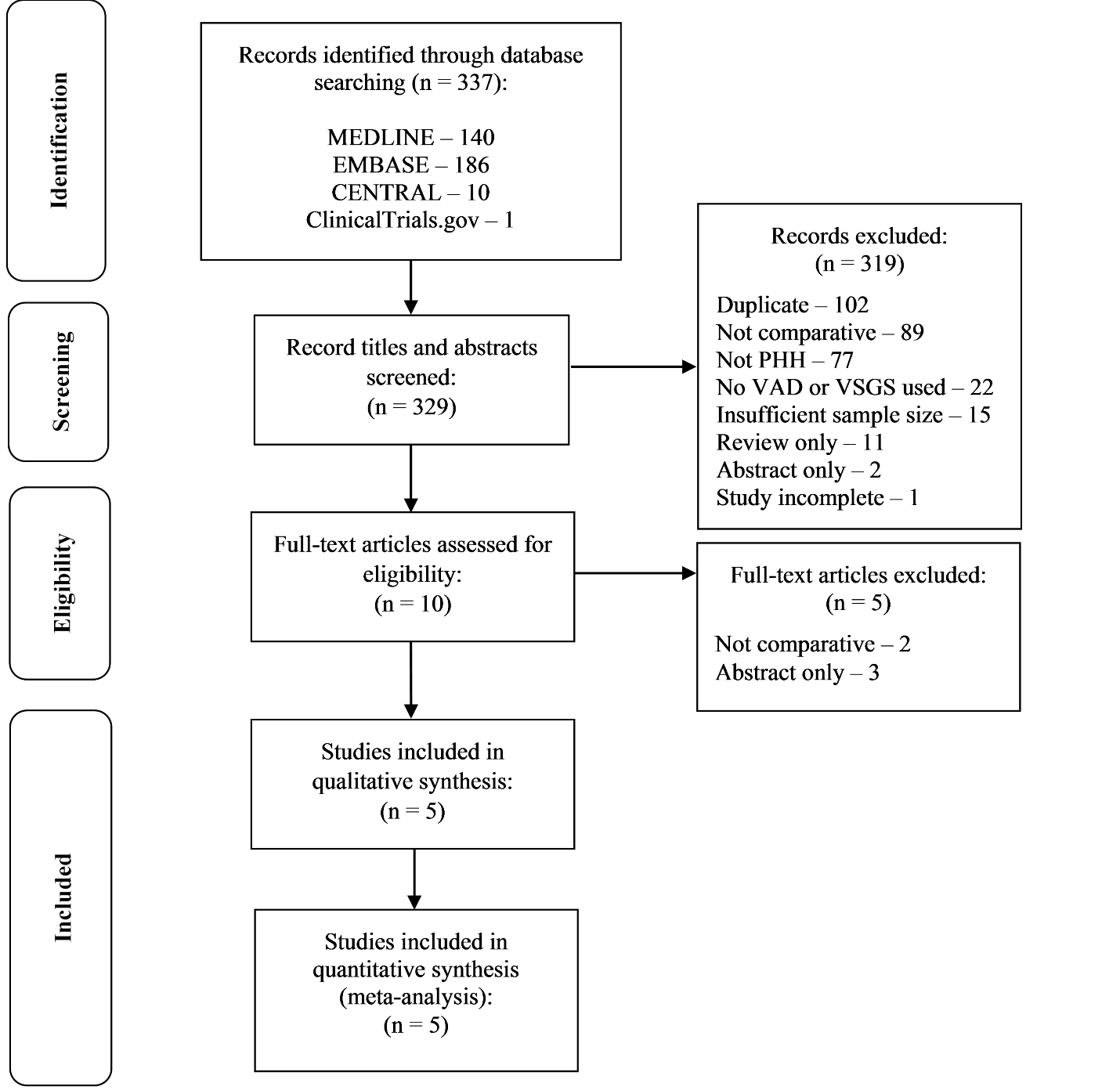

Supplement: Supplementary file 3 — (DOCX 406 kb) [file 381_2015_2951_MOESM3_ESM.docx]
